# The Impact of T-2 Toxin on Vasoactive Intestinal Polypeptide-Like Immunoreactive (VIP-LI) Nerve Structures in the Wall of the Porcine Stomach and Duodenum

**DOI:** 10.3390/toxins10040138

**Published:** 2018-03-26

**Authors:** Krystyna Makowska, Kazimierz Obremski, Slawomir Gonkowski

**Affiliations:** 1 Department of Clinical Physiology, Faculty of Veterinary Medicine, University of Warmia and Mazury, Oczapowskiego Str. 13, 10-718 Olsztyn, Poland; krystyna.makowska@uwm.edu.pl; 2 Department of Veterinary Prevention and Feed Hygiene, Faculty of Veterinary Medicine, University of Warmia and Mazury in Olsztyn, Oczapowskiego Str. 13, 10-718 Olsztyn, Poland; kazimierz.obremski@uwm.edu.pl

**Keywords:** T-2 toxin, enteric nervous system, pig, vasoactive intestinal polypeptide

## Abstract

T-2 toxin is a secondary metabolite of some Fusarium species. It is well-known that this substance can harmfully impact living organisms. Among others, thanks to the ability of crossing the blood–brain barrier, T-2 toxin can affect the central nervous system. Mycotoxins mostly get into the organism through the digestive tract; therefore, first of all they have to break the intestinal barrier, wherein the important component is the enteric nervous system (ENS). However, knowledge about the impact of T-2 toxin on the ENS is rather scant. As a result of the influence of various physiological and pathological agents, ENS can undergo adaptive and reparative processes which manifest as changes in the immunoreactivity of perikaryons for neuronal active substances. So, the aim of the present investigation was to study how low doses of T-2 toxin affect vasoactive intestinal polypeptide-like immunoreactive (VIP-LI) nervous structures in the ENS of the porcine stomach and duodenum. Obtained results have shown that T-2 toxin causes an percentage increase of VIP-LI nerve cells and nerve fibers in every enteric plexus in both fragments of gastrointestinal tract studied. This shows that even low doses of T-2 toxin can have an influence on living organisms.

## 1. Introduction

Mycotoxins are secondary fungal metabolites that may have a multidirectional negative impact on living organisms. Depending on the type of toxic substance, they may show carcinogenic, mutagenic, allergenic, estrogenic, and/or neurotoxic activities [1,2,3,4]. Moreover, due to their widespread presence in the environment, mycotoxins cause not only acute, but also chronic poisoning. Another dangerous consequence of long or intense exposure to these secondary fungal metabolites is the possibility of kidney and/or liver damage resulting in their failure [3,5,6].

Among a broad range of mycotoxins, one of the more dangerous is T-2 toxin, which is synthetized by fungi of the genus Fusarium, namely *Fusarium sporotrichioides*, *langsethiae*, *acuminatum*, and *poae*, and occurs ubiquitously in various food products of vegetable origin, including wheat, sorghum, rye, corn, oats, and rice [2,4,7].

T-2 toxin mostly exhibits cytotoxic and immunosuppressive activities [4,8]. Till now, it has been established that this mycotoxin contributes to such gastrointestinal disorders as alimentary toxic aleukia (ATA) and inflammatory bowel disease [7,9]. Moreover, T-2 toxin can cause thymus and spleen impairments [7,8]. However, one of the most dangerous effects of this toxin is the neurologic toxicity caused by its ability to cross the blood–brain barrier, which results in changes within the central nervous system [4,10]. Because of the above-mentioned negative effects, a high resistance to temperature, and its widespread occurrence in food, T-2 toxin constitutes a serious threat to the health and life of both humans and animals [3,6].

Due to the fact that mycotoxins mostly enter into the organism through the digestive tract, first of all they have to break the intestinal barrier, wherein the important component is the enteric nervous system (ENS). The ENS is situated within the digestive tract, from the esophagus to the rectum, in all mammal species and regulates most stomach and intestine activities. The structure of the ENS varies depending on both the animal species as well as the part of the gastrointestinal (GI) tract. Within the stomach of large mammals, including pigs, the ENS is formed of two intramural ganglionated plexuses: the myenteric plexus (MP), which is situated betwixt the longitudinal and circular muscle layers, and the submucous plexus (SP), which is located between the muscularis mucosa and circular muscle layer [7]. Contrary to the stomach, the ENS in the intestine of big mammals species forms three intramural plexuses: MP, which is situated the same as in the stomach, and two submucous plexuses: the outer submucous plexus (OSP), which is located close to the internal side of the circular muscle layer, and the inner submucous plexus (ISP), which lies between the muscularis mucosa and lamina propria [11,12].

On account of the huge amount of enteric neurons, as well as their significant differentiation and autonomy, the ENS is called the “second” or “intestinal” brain [13]. Because the enteric neurons take part in very different regulatory processes connected with all aspects of intestinal physiology, they may contain a vast number of neuronal active substances [14,15]. Till now, several dozen of such substances have been described. Among them, vasoactive intestinal polypeptide (VIP) deserves special attention. This peptide is known to be a non-noradrenergic, non-cholinergic transmitter (NANC) and the most important inhibitory factor within the GI tract [12,16,17]. First of all, VIP causes the relaxation of gastrointestinal muscles and sphincters [18,19]. Moreover, this substance suppresses secretory activity of the stomach and intestine and dilates blood vessels within the wall of the GI tract and mesentery [20,21]. Some previous studies have revealed the neuroprotective and/or adaptive activity of VIP-positive enteric neurons during pathological processes and after toxic substances administration [22,23,24,25], but these functions of VIP have not been fully elucidated.

On the other hand, although the knowledge about the negative effects of T-2 toxin is not negligible, many aspects connected with the impact of this substance on living organisms still remain unexplored. One of them is the impact of this toxin on the ENS [4,7], which is known to undergo structural, functional, or chemical changes as a result of some physiological and pathological stimuli [26,27,28,29].

So, the present study has been conducted to describe the impact of low doses of T-2 toxin on the distribution of VIP within the enteric perikaryons in the porcine GI tract. Because of the major sensitivity of the ENS to the occurrence of harmful factors in the digestive tract [12,14,26,27,29] and the well-known functions of VIP in intestinal regulatory processes [14,30,31], changes observed during investigations can signify the first subclinical signs of the damage effected by T-2 toxin. The obtained results on the one side can result in a better understanding of the mechanisms connected with the impact of this mycotoxin on living organisms, and on the other side can contribute to an explanation of the roles of VIP during intoxications.

Moreover, the choosing the domestic pig as the laboratory mammal in the investigation allows us to estimate the changes which can occur under the impact of low doses of T-2 toxin in the human enteric nervous system. This assumption is in agreement with previous studies where the neurochemical, histological, and physiological similarities between the human and porcine ENS have been described [12,29,32]. For this reason, the domestic pig seems to be a better animal model for investigations on pathological changes in the human ENS than rodents, which are commonly used for this purpose.

## 2. Results

During the present investigation, no clinical symptoms were observed after T2 toxin administration. Moreover, there were no differences in the food intake between control and experimental groups.

**Figure 1:**
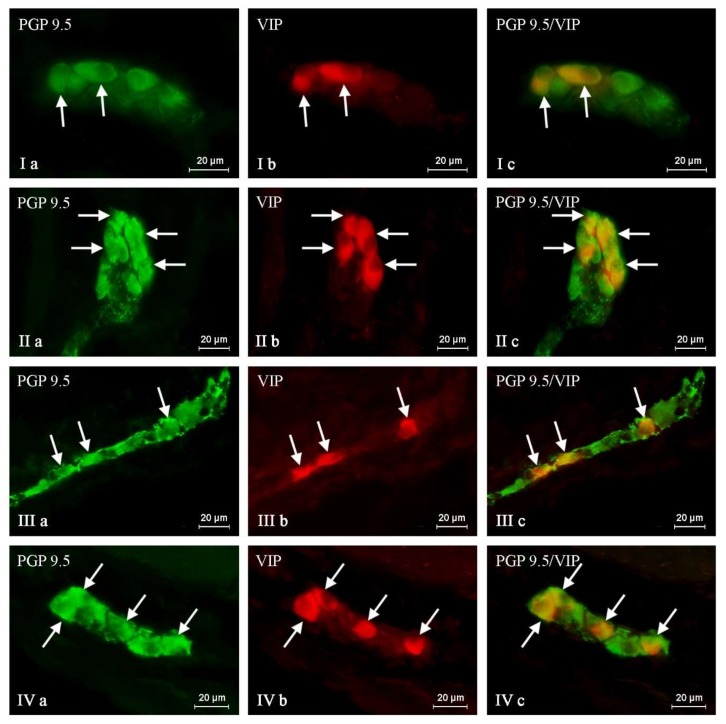
Nerve cell bodies containing the protein gene-product 9.5 (PGP 9.5) pan-neuronal marker (**a**) and vasoactive intestinal polypeptide (VIP) (**b**) within the porcine gastric wall in control animals (I, III) and after T-2 toxin giving (II, IV). I, II: submucous plexus; III, IV: myenteric plexus. Arrows indicate VIP-positive neurons. Photos Ic, IIc, IIIc, and IVc came from the merger of green (PGP 9.5) and red (VIP) fluorescent channels. The pictures were captured with an Olympus XM10 camera.

**Figure 2:**
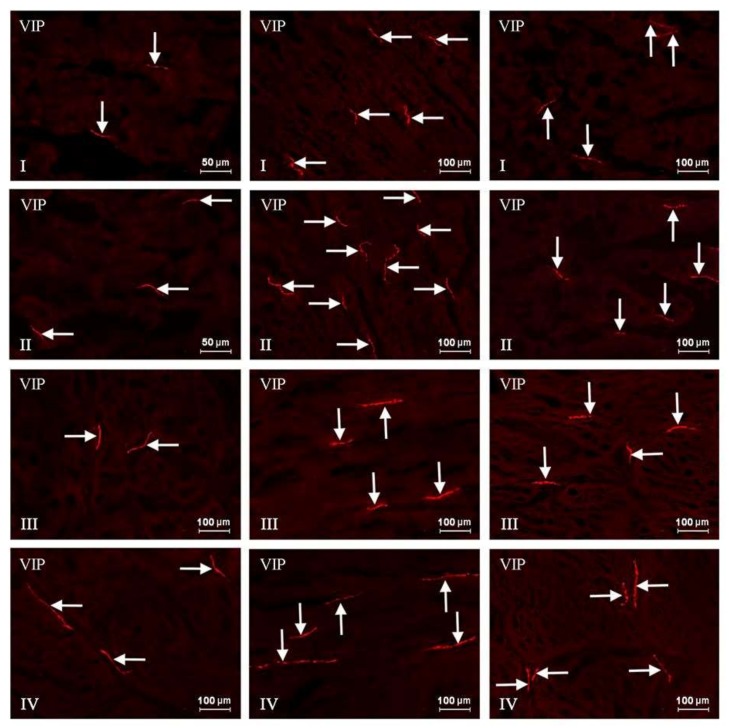
Vasoactive intestinal polypeptide (VIP)-positive nerve fibers in the porcine stomach in control animals (I, III) and after T-2 toxin giving (II, IV). I, II: mucosal layer; III, IV: muscle layer. VIP-positive nerve fibers are indicated by arrows. The pictures were captured with an Olympus XM10 camera.

**Figure 3:**
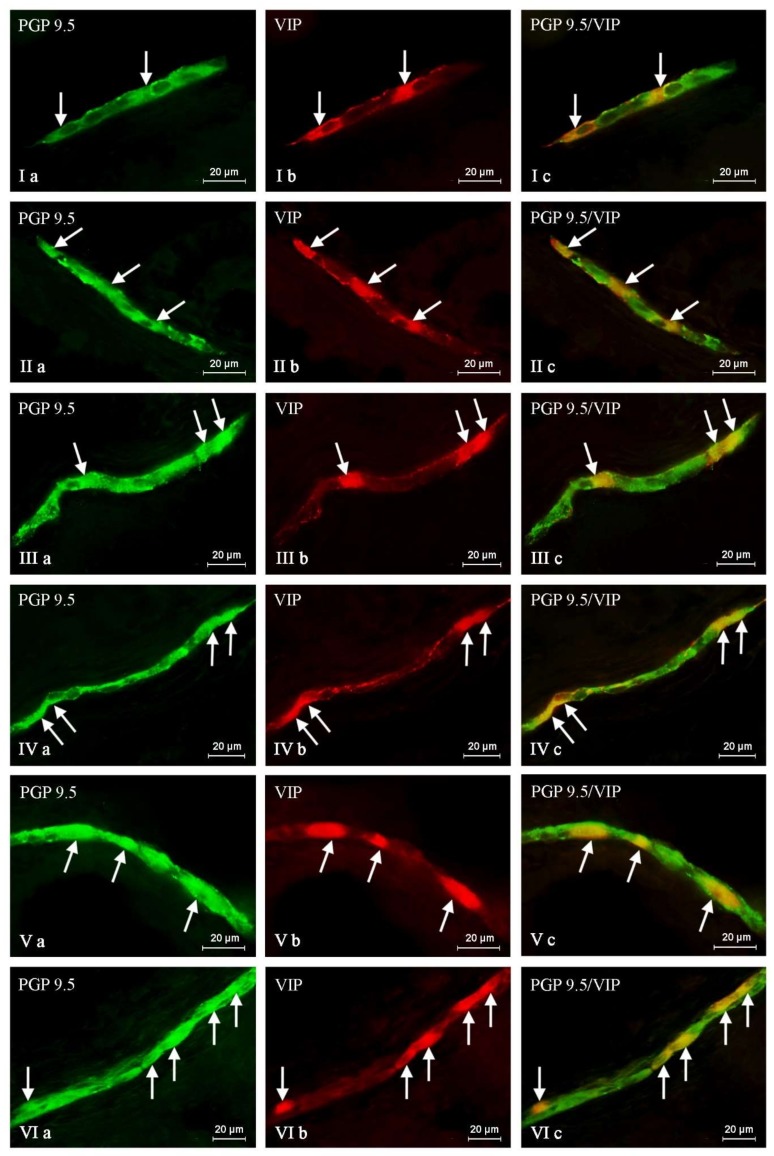
Nerve cell bodies containing the protein gene-product 9.5 (PGP 9.5) pan-neuronal marker (**a**) and vasoactive intestinal polypeptide (VIP) (**b**) within the porcine duodenal wall in control animals (I, III) and after T-2 toxin giving (II, IV). I, II: submucous plexus; III, IV: myenteric plexus. Arrows indicate VIP-positive neurons. Photos Ic, IIc, IIIc, and IVc came from the merger of green (PGP 9.5) and red (VIP) fluorescent channels. The pictures were captured with an Olympus XM10 camera.

**Figure 4:**
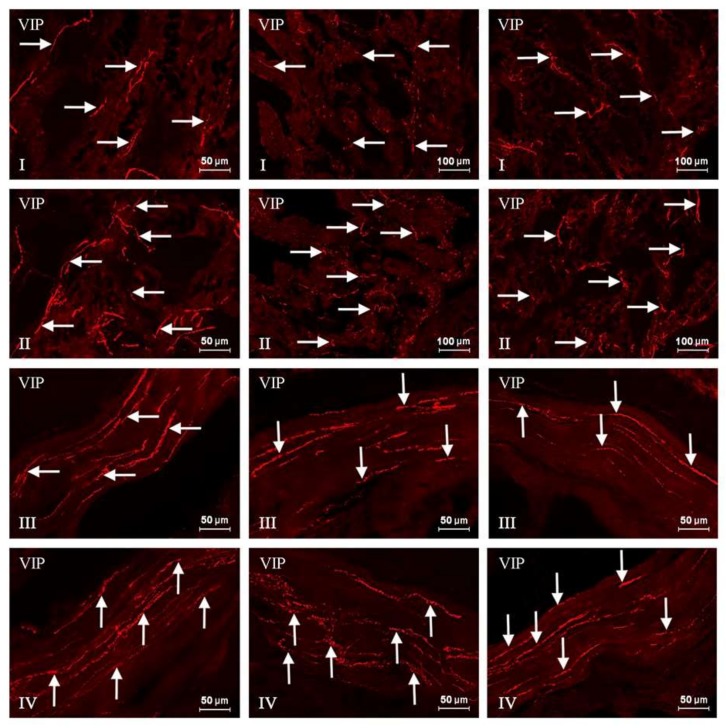
Vasoactive intestinal polypeptide (VIP)-positive nerve fibers in the porcine duodenum in control animals (I, III) and after T-2 toxin giving (II, IV). I, II: mucosal layer; III, IV: muscle layer. VIP-positive nerve fibers are indicated by arrows. The pictures were captured with an Olympus XM10 camera.

In this experiment, VIP-positive neurons and nerve fibers were observed in the ENS of the stomach and the duodenum in both control as well as experimental groups of animals. The number of these structures fluctuated depending on the part of the ENS and the fragment of digestive tract studied (Table 1 and Table 2, Figure 1, Figure 2, Figure 3 and Figure 4).

The percentage of vasoactive intestinal polypeptide-like immunoreactive (VIP-LI) neurons with regard to the number of protein gene-products 9.5 (PGP 9.5) LI cells in both the fundus of the stomach as well as the duodenum of control pigs was substantial. However, in the stomach of C group animals this number was higher and amounted to 37.56 ± 0.84% in the muscular plexus and 36.78 ± 0.4% in the submucous plexus (Figure 1, Table 1). In the same GI tract fragment, unlike nerve cells, the number of VIP-positive nerves was very slight. There has been observed only a single intraganglionic VIP-LI nerve in the MP, whereas such nerves were absent in most of the SP in the stomach of pigs under physiological conditions. Similarly, in case of the fibers situated in the stomachic wall, the number of these neuronal structures was also low and reached only 4.26 ± 0.21 per observation field in the circular muscle layer and 2.84 ± 0.07 in the submucous/mucous layer (Figure 2, Table 1).

In the duodenum from C group, the number of VIP-LI nerve cells was slightly lower than in the case of the stomach. The percentage of these nerve cells amounted to 31.45 ± 0.77%, 32.43 ± 1.83%, and 28.50 ± 1.17% in the MP, OSP, and ISP, respectively (Figure 3, Table 2). Although the number of VIP-positive neurons was comparable in the both GI tract parts studied, the amount of VIP-positive nerve fibers was completely different. The number of intraganglionic nerves was higher than in the stomach, and contrary to the gastric SP, in the submucous plexuses of the duodenum were observed single (in the ISP) and rare (in the OSP) VIP-LI nerve fibers. However, the main difference concerned nerve fibers located in the wall of the studied organs. In contrast to a few VIP-positive nerves in the gastric wall, in the duodenum their number was high and amounted to 17.08 ± 0.08 per observation field in the circular muscle and 32.35 ± 0.32 in the submucous/mucous layers (Figure 4, Table 2).

Treatment with T-2 toxin caused changes in the percentage of VIP-LI enteric perikaryons and the density of nerve fibers positive for the studied substance. These alterations consisted mainly in an increase in the number of VIP-positive nerve structures. However, the intensity of these deviations clearly depended on both the GI tract fragment and part of the ENS studied. The biggest changes concerned the MP and OSP in the duodenum, where the percentage of VIP-positive perikaryons increased about eight percentage points (pp). Considerably lower alterations were observed in the duodenal inner submucous plexus (about a seven pp increase) (Figure 3, Table 2). Similar changes have been noted in the gastric MP (increase of five pp) and SP (increase of six pp) (Figure 1, Table 1). In the experimental group, the concentration of intraganglionic VIP-positive nerves was also greater than in the C group; however, these alterations were not so significant and were noted in the stomachic SP and the duodenal MP and ISP. Furthermore, marked changes in the density of VIP-LI nerves in the muscle and mucous layers were noted in the T2 group. The clearest increase in the number of nerves per observation field was noted in the duodenal submucous/mucous layer: from 32.35 ± 0.32 to 39.97 ± 1.23, whereas in the stomach, these values accelerated from 2.84 ± 0.07 to 4.15 ± 0.15. Alterations in the amount of VIP-LI nerve fibers in the muscle layer were less observable and reached about 5.17 ± 0.11 in the duodenum and about 21.22 ± 0.24 within the stomach (Table 1 and Table 2, Figure 2 and Figure 4).

## 3. Discussion

The present observations have confirmed that VIP is present in the enteric neurons and nerve fibers of the porcine stomach and duodenum. It is compatible with previous investigations describing this substance as an important neurotransmitter in the ENS of a wide range of mammals species, including humans [22,24,31,33,34]. It is known that enteric neurons capable of VIP synthesis first of all have inhibitory effects and their stimulation causes the relaxation of the intestinal smooth muscles and a decrease in the secretory activity of the digestive tract. Inhibitory effects on gastrointestinal activities are not the only functions of VIP, because this substance also takes part in the regulation of immune processes [35,36] and intestinal blood flow [12,37] as well as may influence intestinal commensal and pathogenic bacteria populations [38]. Moreover, some studies have described the presence of VIP in enteric sensory neurons [39]. In spite of the relatively numerous studies concerning VIP in the GI tract, some aspects connected with the functions of this peptide remain unexplained. One of them is the physiological factors that influence the number of VIP-positive enteric neurons. The percentage of neurons immunoreactive to VIP observed in control animals during the present study differ from the values described in previous studies [22,24,31,33,34], which suggests that the synthesis of this peptide by enteric neuronal cells depends on unidentified stimuli that would probably include (apart from individual variations) type of diet, farming conditions, and/or environmental factors. Moreover, differences in the number of VIP-LI enteric nerve cells between the stomach and duodenum observed during the present experiment strongly imply that the precise roles of VIP in the digestive tract vary depending on the part of the GI tract studied.

During the present study, the impact of low doses of T-2 toxin on the number of neurons immunoreactive to VIP was noted in all parts of the ENS within the stomach and duodenum. Due to the fact that knowledge about the impact of T-2 toxin on the ENS is extremely scant, and VIP may have multidirectional functions in enteric neurons, the exact elucidation of the observed fluctuations is rather difficult. Moreover, observed changes may arise from various processes, including disorders in the transcription and/or translation stage of VIP synthesis as well as from disturbance of the peptide inside neuronal cells.

These changes are probably connected with the neurotoxic effects of this mycotoxin, which are the result of reactions of epoxides included in the molecules of the described substance with DNA and proteins of neuronal cells [40]. Furthermore, T-2 toxin is a commonly known inhibitor of cellular protein synthesis and a factor causing changes in the metabolism of membrane phospholipids [41,42]. Admittedly, the knowledge concerning T-2 toxin-induced changes in the ENS is extremely scant. Namely, our previous observations have shown that even low dosages of this mycotoxin may influence the number of enteric neurons immunoreactive to cocaine- and amphetamine-regulated transcripts in various parts of the GI tract [7] as well as affect the neurochemical characterization of calcitonin gene-related peptide-positive structures of the ENS within the descending colon [4]. Nevertheless, the mechanisms underlying the above-mentioned fluctuations in chemical coding of the enteric neurons remain unknown. On the other hand, it can be expected that processes connected with the neurotoxicity of T-2 toxin in the ENS are similar to those observed in the other parts of the nervous system. According to previous investigations, it is known that T-2 toxin causes alterations in the levels of brain biogenic monoamines [43] and induces apoptosis in brain neuronal cells [44]. The reason of the exacerbation of apoptotic reactions in the nervous tissue is probably connected with dysfunction of mitochondria caused by a T-2 toxin-induced decrease in the levels of mitochondrial NADH-dehydrogenase and cytochrome oxidase enzymes occurring in mitochondria-related genes [45]. The thesis about connections of observed changes with neurotoxic effects of T-2 toxin is strongly supported by the fact that VIP is known to be an important neuroprotective factor. Some previous works have described that this peptide has multidirectional neurotrophic activities. Among others, VIP stimulates mitosis within the astrocytes [46], supports neuronal differentiation of embryonic stem cells [47], and increases neuronal survival under various pathological factors [48]. Furthermore, it is known that VIP has neuroprotective functions during excitotoxicity, i.e., pathological processes consisting in neuronal cell damage by excessive stimulation by some neurotransmitters [49], and therefore inhibits the lesion of neurons in the brain cortex [50]. The neuroprotective effects of VIP are probably connected with direct actions on microglial cells, which are the main source of inflammatory factors damaging neurons in the central nervous system [51,52]. The above-mentioned VIP properties have caused this substance as well as its analogs to be tested as drugs to treat neurodegenerative diseases [48]. Contrary to the central nervous system, knowledge about the neuroprotective roles of VIP within the enteric nervous system is more limited. Nevertheless, the previous investigations have shown that this substance increases the survival of cultured enteric nerve cells undergoing some pathological factors, including axotomy or bacterial lipopolysaccharide [23,53]. In view of the foregoing, it is very likely that dissimilarities in VIP-LI observed during the present investigation are an effect of neurotoxic activity of T-2 and are the result of neuroprotective and/or adaptive processes within the ENS, which aim to maintain homeostasis in the GI tract under the influence of the toxin.

The other mechanisms connected with observed changes may result from pro-inflammatory and damaging properties of T-2. T-2 toxin may cause multidirectional disorders, among which is alimentary toxic aleukia (ATA). During these processes, inflammatory changes and GI tract injuries occur, which result in intestinal epithelial barrier disruption and manifest by vomiting, nausea, and diarrhea [2,54]. It should be pointed out that inflammatory changes in the intestine have been also observed after relatively low doses of T-2 toxin administration [55]. These changes may influence indirectly the ENS. This is all the more likely as the interdependences between intestinal epithelial cells and the ENS are commonly known [56]. Moreover, VIP is one of the factors that is are involved in gastrointestinal inflammatory processes, and enteric neurons respond to these processes with the growth of VIP expression [22,23,24,36,37].

Changes in VIP expression observed during this investigation can be also caused by the stimulation of the gastrointestinal immune system, because this substance is considered to be a major immunoregulatory neuropeptide. Previous investigations have described VIP as an anti-inflammatory factor, which first of all, through the VPAC1 type of receptor, inhibits macrophage activity [57]. These effects on the one side lead to a decrease in the synthesis of pro-inflammatory cytokines, including TNFα, IL-6, and IL-12, and on the other side result in a simultaneous increase in the levels of IL-10, which is the main anti-inflammatory factor [57,58]. Moreover, it is known that VIP may affect the phagocytosis, migration, and adherence of macrophages, but the exact mechanisms connected with these activities have not been fully elucidated. Some studies have described that VIP stimulates the above-mentioned processes, whereas other investigations have reported inhibitory effects [59]. The described polypeptide also inhibits the synthesis and activity of the inducible isoform of nitric oxide synthase (iNOS) within immunological cells [57].

The second type of immune cells that may be subject to the influence of VIP are lymphocytes. Previous studies have reported that this polypeptide influences Th1/Th2 cells’ balance by the inhibition of Th1 cells, the support of Th2 cells [57], and the reduction of T cells migration through intestinal Peyer’s patches [59]. Moreover, as in the case of macrophages, VIP changes the levels of cytokines synthesized in Th1 and Th2 cells. For example, VIP suppresses the production of IL-2 but increases IL-5 synthesis [59]. The above-mentioned immunoregulatory functions of VIP have made this polypeptide a potential therapeutic agent during various inflammatory and autoimmune diseases, including lupus, autoimmune thyroiditis, and arthritis [57]. The hypothesis that fluctuations in the VIP-like immunoreactivity observed during the present investigation are the result of the cooperation of the ENS with the gastrointestinal immune system and may be the answer of the enteric nervous structures to the inflammatory processes induced by T-2 toxin is probable in the light of previous studies. These studies have shown the described mycotoxin to have an influence on the number of types of lymphocytes and the expression of cytokines within the porcine ileal Peyer’s patches [60]. Since during the present study low doses of T-2 toxin have been used, and symptoms of inflammation in the experimental animals were not observed, it cannot be excluded that the observed fluctuations were the first sign of a subclinical inflammatory process.

## 4. Conclusions

The obtained results have shown that even low doses of T-2 toxin are not neutral for mammal organisms and may change the chemical coding of the neurons and nerve fibers in the stomach and duodenum. Moreover, they confirm the important roles of VIP within the ENS under physiological conditions and after administration of T-2 toxin. The fluctuations of VIP-like immunoreactivity observed during the study are probably connected with neuroprotective functions of this substance and/or its participation in immune processes. Nevertheless, due to the multidirectional negative activities of T-2 toxin and the various functions of VIP within the digestive tract, the exact elucidation of mechanisms taking part in observed changes remains for further studies.

## 5. Materials and Methods

The experiment was performed on 10 immature female pigs of the Large White Polish breed (8 weeks old, 20 kg body weight (b.w.)). Both the division of animals into groups as well as doses of T-2 toxin were performed as described previously by Makowska et al. [4]. In short, after a five-day adaptation period, animals were randomly divided into two equal groups (five animals in each). Animals of both groups received capsules per os, once a day for 42 days. In the control group (C group), the administrated capsules were empty, while in the experimental group (T2 group), capsules contained T-2 toxin (12 µg/kg b.w. per day).

All animals were fed with feed appropriate for age and species “WIGOR 3” (WIPASZ S.A, Olsztyn, Poland) of known composition, which is available on the producer’s website. Furthermore, to eliminate additional mycotoxins contamination of the feed, the presence of Aflatoxin B1, T-2 toxin, ochratoxin A (OTA ), ZEN, alpha-zearalenol (α-ZEL), and deoxynivalenol (DON) were evaluated using common separation techniques with immunoaffinity columns (Afla-TestR P Aflatoxin testing system, G1010, VICAM, Watertown, MA, USA; T-2-TestTM HPLC Mycotoxin Testing System G1028, VICAM, Watertown, MA, USA; Ochra-TestTM WB Mycotoxin Testing System, G1033, VICAM, Watertown, MA, USA; Zearala-Test™ Zearalenone Testing System, G1012, VICAM, Watertown, MA, USA; DON-Test™ DON Testing System, VICAM, Watertown, MA, USA) and high performance liquid chromatography (HPLC) (Hewlett Packard, type 1050 and 1100) with fluorescent and/or UV detection methods. All of the above-mentioned mycotoxins were absent in the studied feed.

Throughout the duration of the investigation, gilts stayed under standard experimental conditions and all procedures were conducted in agreement with the directions of the Local Ethical Committee for Animal Experiments in Olsztyn (Poland) (decision from 28 November 2012, No. 73/2012/DTN).

After 42 days, animals from both groups were premedicated with Stressnil (Janssen, Beerse, Belgium, 75 μL/kg b.w.) then, after 15 min, euthanized with high doses of sodium thiopental (Thiopental, Sandoz, Kundl-Rakúsko, Austria). Directly after euthanasia, the fragments (with a length of about 2 cm) of the stomach fundus (20 cm before the pylorus) and duodenum (5 cm after the pylorus) were collected from all pigs and fixed for one hour in a solution of 4% buffered paraformaldehyde (pH 7.4). Afterward, the taken fragments of the stomach and duodenum were put in phosphate buffer (0.1 M, pH 7.4, at 4 °C). This procedure lasted for three days with a daily exchange of the buffer. Later on, the tissues were inserted into 18% phosphate-buffered sucrose and storage for 3 weeks at 4 °C. Then, after freezing at −22 °C, the tissue fragments of both stomach and duodenum were cut perpendicular to the lumen of the digestive tract into 14-µm-thick sections with freezing microtome (Microm, HM 525, Walldorf, Germany).

Such prepared tissue fragments were subjected to a routine double-labelling immunofluorescence method as previously described by Gonkowski 2013 [29].

In brief, the immunofluorescence labelling was carried out as follows. Frozen fragments of the stomach and duodenum were dried (45 min, room temperature (rt)) and incubated with blocking solution (10% goat serum, 0.1% bovine serum albumin (BSA), 0.01% NaN3, Triton X-100, and thimerosal in PBS) for 1 h at rt. Next, fragments of the GI tract were incubated with a mixture of two antibodies: mouse monoclonal antibody directed towards protein gene-product 9.5 (PGP 9.5, Biogenesis, UK, catalogue No. 7863-2004, working dilution 1:2000, used here as a pan-neuronal marker) and rabbit polyclonal anti-VIP antibody (Cappel, Aurora, OH, USA, catalogue No. 11428, working dilution 1:5000). For the incubation process, slices covered with antibodies mixture were left overnight at rt in a humid chamber. The next day, for visualization of the primary antibodies connected with suitable antigens, an incubation with the mixture of species-specific secondary antisera conjugated to selected fluorochromes (Alexa fluor 488 donkey anti-mouse IgG and Alexa fluor 546 donkey anti-rabbit IgG, both antibodies from Invitrogen, Carlsbad, CA, USA, working dilution 1:1000) has been performed. This incubation process lasted 1 h at rt. After every stage of the immunofluorescence method, samples were rinsed with PBS (3 × 15 min, pH 7.4).

During the present investigation, routine specificity tests of antibodies were performed. Pre-absorption of the antibodies with appropriate antigens and omission and replacement tests were performed to eliminate non-specific labelling.

To evaluate the percentage of VIP-like immunoreactive perikaryons in relation to all enteric neurons, no less than 500 cells labelled with PGP-9.5 (treated as 100%) in the particular type of enteric plexus from each part of the digestive tract and each animal were examined for the presence of VIP. Only nerve cells with clearly visible nuclei were considered in the present study. The received results were pooled and presented as mean ± SEM. To obviate double counting of the same perikaryons, the investigated sections of the GI tract were located no less than 150 µm apart.

To define the density of intraganglionic nerves positive for VIP, an arbitrary scale was used. In this scale, (-) indicated the absence of nerve fibers, (+) single fibers, (++) rare fibers, (+++) a dense network of fibers, and (++++) a very dense meshwork of VIP-LI fibers. The evaluation of VIP-LI nerve fibers in the muscular and mucosal layers in the stomach and duodenum was carried out on the basis of the counting of all VIP-positive nerves per area studied (0.1 mm^2^). Such evaluation was carried out in four sections per animal (in five fields per section) and the obtained results were presented as a mean ± SEM.

All indications during the present study were performed using an Olympus BX51 microscope with epi-fluorescence and appropriate filter sets connected with an Olympus XM10 camera.

Statistical analysis was performed with Student’s *t*-test (Statistica 12, StatSoft, Inc., Cracow, Poland). The differences were considered statistically significant at *p* <0.05.

## Figures and Tables

**Table 1:** Vasoactive intestinal polypeptide (VIP)-positive nerve cells and fibers in the stomach of the pig. CML: circular muscle layer; MP: myenteric plexus; CB: cell bodies; NF: nerve fibers; SP: submucous plexus; S/ML: submucosal/mucosal layer.

STOMACH		Group C	Group T2
CML ^1^		4.26 ± 0.21	5.17 ± 0.11 *
MP	CB ^2^	37.56± 0.84	42.85 ± 0.74 *
	NF ^3^	+	+
SP	CB ^2^	36.78 ± 0.4	43.83 ± 1.18 *
	NF ^3^	−	+
S/ML ^1^		2.84 ± 0.07	4.15 ± 0.15 *

^1^ Mean number of VIP-positive nerves per microscopic field (mean ± standard error of the mean (SEM)); ^2^ The percent of VIP-positive nerve cells (mean ± SEM) in regard to perikarya immunoreactive to protein gene-product 9.5 (PGP 9.5) (pan neuronal marker); ^3^ the consistence of intraganglionic VIP-positive fibers shown in mean units on the scale from (-), presenting the absence of VIP-positive nerves, to (++++), indicating a very dense meshwork of fibers studied. Statistically significant differences (*p* ≤ 0.05) are marked with *.

**Table 2:** VIP-positive nerve cells and fibers in the duodenum of the pig. CML: circular muscle layer; MP: myenteric plexus; CB: cell bodies; NF: nerve fibers; OSP: outer submucous plexus; ISP: inner submucous plexus; S/ML: submucosal/mucosal layer.

DUDODENUM		Group C	Group T2
CML ^1^		17.08 ± 0.08	21.22 ± 0.24 *
MP	CB ^2^	31.45 ± 0.77	39.24 ± 1.02 *
	NF ^3^	+	++
OSP	CB ^2^	32.43 ± 1.83	40.59 ± 0.67 *
	NF ^3^	++	++
ISP	CB ^2^	28.50 ± 1.17	35.42 ± 1.52 *
	NF ^3^	+	++
S/ML ^1^		32.35 ± 0.32	39.97 ± 1.23 *

^1^ Mean number of VIP-positive nerves per microscopic field (mean ± SEM); ^2^ The percent of VIP-positive nerve cells (mean ± SEM) in regard to perikarya immunoreactive for PGP 9.5 (pan neuronal marker); ^3^ the consistence of intraganglionic VIP-positive fibers shown in mean units on the scale from (-), presenting the absence of VIP-positive nerves, to (++++), indicating a very dense meshwork of fibers studied. Statistically significant differences (*p* ≤ 0.05) are marked with *.
